# The Potential of Lung Epithelium Specific Proteins as Biomarkers for COVID-19-Associated Lung Injury

**DOI:** 10.3390/diagnostics11091643

**Published:** 2021-09-08

**Authors:** Sultan Almuntashiri, Chelsea James, Xiaoyun Wang, Budder Siddiqui, Duo Zhang

**Affiliations:** 1 Clinical and Experimental Therapeutics, College of Pharmacy, University of Georgia and Charlie Norwood VA Medical Center, Augusta, GA 30912, USA; salmuntashiri@augusta.edu (S.A.); xiaoyunwang@uga.edu (X.W.); 2 Department of Clinical Pharmacy, College of Pharmacy, University of Hail, Hail 55473, Saudi Arabia; 3 College of Pharmacy, University of Georgia, Augusta, GA 30912, USA; chelsea.james@uga.edu; 4 Division of Infectious Diseases, Medical College of Georgia, Augusta University, Augusta, GA 30912, USA; bsiddiqui@augusta.edu; 5 Vascular Biology Center, Augusta University, Augusta, GA 30912, USA

**Keywords:** SARS-CoV, SARS-CoV-2, KL-6, inflammation, epithelial cell, CC16, surfactant proteins

## Abstract

Severe Acute Respiratory Syndrome Coronavirus 2 (SARS-CoV-2) infection was first reported in Wuhan, China, and was declared a pandemic by the World Health Organization (WHO) on 20 March 2020. The respiratory system is the major organ system affected by COVID-19. Numerous studies have found lung abnormalities in patients with COVID-19, including shortness of breath, respiratory failure, and acute respiratory distress syndrome. The identification of lung-specific biomarkers that are easily measurable in serum would be valuable for both clinicians and patients with such conditions. This review is focused on the pneumoproteins and their potential to serve as biomarkers for COVID-19-associated lung injury, including Krebs von den Lungen-6 (KL-6), surfactant proteins (SP-A, SP-B, SP-C, SP-D), and Clara cell secretory protein (CC16). The current findings indicate the aforementioned pneumoproteins may reflect the severity of pulmonary manifestations and could serve as potential biomarkers in COVID-19-related lung injury.

## 1. Introduction

Severe Acute Respiratory Syndrome Coronavirus 2 (SARS-CoV-2) infection was first reported in December 2019, in Wuhan, China, and this outbreak was declared a pandemic by the World Health Organization (WHO) in March 2020 [1,2]. SARS-CoV-2 has infected more than 100 million people. As of 2 June 2021, the WHO has reported 3,840,223 deaths as a result of SARS-CoV-2. Coronaviruses are a large group of enveloped, single-stranded RNA viruses [3]. In the past two decades, two coronaviruses, Severe Acute Respiratory Syndrome Coronavirus (SARS-CoV) and Middle East Respiratory Syndrome Coronavirus (MERS-CoV), have emerged and affected public health in terms of morbidity and mortality. Globally, SARS-CoV has infected over 8,000 people with a mortality rate of around 10%, while MERS-CoV has infected 857 patients with a mortality rate of 35.5% [4,5].

SARS-CoV-2 is primarily transmitted by droplets from sneezes or coughs of symptomatic and asymptomatic infected patients [6,7]. The clinical features of coronavirus disease 2019 (COVID-19) range from asymptomatic to critical. Patients in critical conditions require invasive or non-invasive oxygen therapy [8,9]. The respiratory system is the major organ system affected by COVID-19. Various studies have found lung abnormalities in patients with COVID-19, including shortness of breath, respiratory failure, and acute respiratory distress syndrome (ARDS) [10,11]. It has previously been reported that approximately 30% of patients with SARS and MERS had persisting pulmonary manifestations following recovery [12,13,14]. Thus, the identification of lung epithelium-specific proteins that are easily measurable in the circulation would be beneficial in monitoring the lung conditions of such patients. In this review, we highlight the role of pneumoproteins, namely Krebs von den Lungen-6 (KL-6), surfactant proteins (SP-A, SP-B, SP-C, SP-D), and Clara cell secretory protein (CC16), as potential pulmonary biomarkers for COVID-19-associated lung injury.

## 2. Krebs von den Lungen-6 (KL-6) in COVID-19 Patients

Krebs von den Lungen-6 (KL-6) is a high-molecular-weight glycoprotein mainly produced by damaged or regenerating type II pneumocytes and bronchial epithelial cells [15]. It plays a major role in fibroblast stimulation and apoptosis inhibition in normal lungs [16]. KL-6 promotes chemotactic activity and anti-apoptotic effects on human lung fibroblasts, but the exact mechanism remains unknown [16]. Fibroblast apoptosis is vital to normal wound healing, whereas apoptosis resistance in fibroblasts leads to progressive scarring and pulmonary fibrosis [17]. Since KL-6 has anti-apoptotic activities on stimulated fibroblasts, an elevated serum level of KL-6 is known to be a sign of active pulmonary fibrosis. KL-6 levels are raised in the serum of patients with interstitial lung diseases (ILDs), ARDS, pulmonary tuberculosis, and bronchopulmonary dysplasia (BPD) [18,19,20,21,22,23]. In COVID-19, it is suspected that viruses can induce cytopathic effects against type I or II pneumocytes and contribute to lung injury [24]. It is also postulated the epithelial cell injuries cause leakage in the air-blood barrier, leading to an elevation in the serum levels of KL-6 [21]. Measuring KL-6 in serum could evaluate lung scarring and aid in diagnosis and prognosis during or after COVID-19 infection.

The first study that measured KL-6 levels from COVID-19 patients found that the serum concentrations of KL-6 were significantly higher in severe cases than in the non-severe group. The high levels of KL-6 detected in patients with severe pulmonary involvement suggests that KL-6 levels in serum can be a useful measurement for assessing the interstitial lung damage, epithelial lung alterations, and remodeling processes after SARS-CoV-2 infection [25]. Similarly, another study reported that KL-6 levels were significantly higher in COVID-19 patient populations than in healthy subjects. Interestingly, when comparing the KL-6 levels from COVID-19 cases to a group of patients with ILDs, KL-6 concentrations were significantly higher in ILDs than in the COVID group, thus concluding that KL-6 can be a potential biomarker to assess the severity of ILDs in patients with COVID-19 [26]. Furthermore, KL-6 levels were significantly elevated in severe COVID-19 patients compared to mild or non-COVID-19 subjects [27]. In the two previous studies, KL-6 was significantly correlated with different oxygenation indices, such as arterial pulse oxygen saturation (SpO_2_), arterial partial pressure of oxygen (PaO_2_), and oxygen partial pressure difference of alveolar artery (PA-aDO_2_), which reflect the severity of COVID-19. Moreover, one study assessed KL-6 levels in COVID-19 patients at two different times: at diagnosis and 1 week later. KL-6 serum levels were significantly higher in the severe subjects compared to the non-severe group. The median differences in KL-6 levels between those different times in each group were 404 U/mL and 0 U/mL in the severe and non-severe groups, respectively (*p* < 0.001) [28]. Thus, it is recommended to measure KL-6 repeatedly to evaluate the potential severity or recovery of COVID-19. In addition, one study aimed to investigate and distinguish between the clinical, imaging, and laboratory findings of COVID-19 cases. The researchers found that patients with CT infiltrate had higher KL-6 levels in comparison to patients with no CT infiltrates [29]. In another recent study, researchers measured KL-6 in COVID-19 patients during hospitalization and 6–9 months after being discharged from the hospital. KL-6 levels were significantly decreased after months of recovery in comparison to KL-6 levels that were obtained at the admission, suggesting that interstitial lung abnormalities related to SARS-CoV-2 infection were not progressive in the majority of patients [30]. Similarly, a recent study reported that KL-6 levels gradually reduced after reaching a peak in 1 month [31].

On the other hand, one study correlated KL-6 levels and the fibrotic changes that were associated with COVID-19 by performing chest CT imaging tests. It was shown that the higher levels of KL-6 in COVID-19 patients represented fibrotic changes [30]. A recent study also identified COVID-19 infection as a potential factor that causes lung fibrosis [32]. Thus, high serum concentrations of KL-6 might predict the pulmonary fibrotic sequelae in COVID-19 patients. Peng et al. found that serum KL-6 levels in COVID-19 patients who developed pulmonary fibrosis were higher than those who did not develop pulmonary fibrosis [33]. In the same study, the prevalence rate of pulmonary fibrosis in severe COVID-19 cases was much higher than in non-severe cases, indicating that high KL-6 levels in serum in severe COVID-19 cases may be a useful biomarker for predicting lung fibrosis [33].

Based on these findings, it is clear that KL-6 levels in serum were higher in most COVID-19 patients, as shown in Table 1. However, it seems that KL-6 increased only in severe cases and not in mild cases. KL-6 levels from mild patients and healthy controls were almost similar, suggesting that not all cases of COVID-19 lead to lung injury. Another finding is that KL-6 was higher in ILDs compared to severe cases of COVID-19, confirming the reliability of KL-6 as a serum assessment of ILDs’ severity and suggesting a promising serum biomarker in the early stages of lung injury in COVID-19. As shown in Table 2 and Table 3, KL-6 levels in serum were different between those studies, and the cutoff values were different as well. Thus, there is a need to establish an optimal reference range to allow clinicians to easily evaluate their patients. In addition, more studies using a larger number of participants with COVID-19 are needed to draw accurate conclusions. In the meantime, it is recommended to measure KL-6 in serum as a lung biomarker in response to SARS-CoV-2 infection.

## 3. Surfactant Proteins A, B, C, and D

Pulmonary surfactants are complex mixtures composed of lipids and proteins that are produced by epithelial type II cells into the alveolar space [40]. The main function of the surfactants is to reduce the surface tension at the air-liquid interface in the alveoli, thereby preventing its collapse at end-expiration [41]. There are four surfactant proteins—SP-A, SP-B, SP-C, and SP-D—and each one has a different biological role. These surfactants proteins can be divided into two groups: hydrophilic and hydrophobic. SP-A and SP-D are hydrophilic proteins, and they are known for their contribution to pulmonary immunity and the regulation of inflammation. The molecular weights for SP-A and SP-D are 36 kDa and 43 kDa, respectively, and they are homologous in their sequences [42]. It has been shown that Clara cells synthesize and release mature SP-A, SP-B, and SP-D proteins [43,44]. However, only epithelial type II cells can produce all the surfactant components, including the four types of surfactant proteins and phospholipids [45,46,47].

SP-B and SP-C are hydrophobic proteins that are essential for the normal functioning of pulmonary surfactants. Their biological roles are to maintain the surfactant structure and to stabilize the lipid layers during each respiratory cycle [48]. SP-B is the smallest surfactant protein, with a molecular weight of around 8 kDa in its mature form. The molecular weight of SP-C is 21 kDa [49,50]. SP-C is secreted by type II pneumocytes as an integral membrane precursor protein. In adult lungs, SP-B mRNA expression is limited to alveolar type II epithelial cells and Clara cells. Similarly, SP-C becomes limited to alveolar type II cells after its expression in the distal epithelium during early lung development [51].

Lung inflammation and injury influence the secretion of these proteins from the lung epithelial cells into the systematic circulation. However, the detection of such proteins in serum may reflect an abnormality in the alveolar epithelial barrier [41]. Thus, they have been examined as biomarkers in pulmonary diseases such as ARDS, chronic obstructive pulmonary disease (COPD), and idiopathic pulmonary fibrosis (IPF) [52,53,54,55].

Regarding coronavirus infections, two studies have measured SP-D after SARS-CoV-1 and SARS-CoV-2 infections using enzyme-linked immunosorbent assays (ELISA). No study has measured other surfactant proteins in the serum during the aforementioned pandemics. SP-D levels were significantly higher in COVID-19 patients than in the control group and were associated with the development of ARDS and macrophage activation syndrome (MAS), both of which are considered critical complications of COVID-19. Furthermore, SP-D levels were elevated in non-survivor patients compared to survivors. The ratio of arterial oxygen partial pressure to the fractional inspired oxygen (PaO_2_/FiO_2_) in hypoxemic patients was significantly correlated to SP-D [38]. Thus, measuring SP-D in serum may be valuable for assessing respiratory complications following SARS-CoV-2 infection. In a SARS-CoV-1 study, SP-D levels in serum were higher in comparison to the healthy controls. In addition, there was a significant correlation between SP-D and SARS-CoV-specific antibodies. Interestingly, SP-D levels were not significantly different between SARS patients and patients with community acquired pneumonia (CAP), suggesting a possibility to consider SP-D as a lung biomarker in response to infectious diseases caused by different pathogens [39].

ELISA data in these two studies indicated that SP-D levels were much higher in SARS-CoV-1 than COVID-19, as shown in Table 2, confirming that SARS was more serious than COVID-19 in morbidity and mortality [56]. The importance of SP-D as a lung biomarker has been tested in preclinical and clinical studies. SP-D is primarily released from the lungs and is increased in circulation due to pulmonary leakage, as reported in experimental studies [57]. In clinical studies, higher levels of SP-D were associated with mortality in patients with acute lung injury (ALI)/ARDS and were correlated with worse clinical outcomes [58,59]. Recently, elevated SP-D was found in patients with COVID-19 infection compared to the control groups. Thus, it is recommended to monitor patients with a possible risk of SARS-CoV-2-associated lung injury by measuring SP-D levels in serum. Future studies may shed light on other surfactant proteins that have not been measured yet or validate the current findings.

## 4. Clara Cell Secretory Protein (CC16) as a Potential Biomarker for COVID-19

Clara cell secretory protein, also known as club cell secretory protein or uteroglobin, is a 10–16 kDa (CC16) protein primarily secreted by the non-ciliated bronchial epithelial cells in the respiratory epithelium. CC16 can be easily detected in the circulatory system under both normal and pathological conditions [60]. This protein appears to have a protective effect against the respiratory inflammatory response by modulating the activities of phospholipases A2, interferon-γ, and tumor necrosis factor-α (TNF-α) [61]. CC16 has been studied as a biomarker for lung epithelial injury in most pulmonary diseases, including COPD, asthma, idiopathic pulmonary fibrosis, ARDS, and sarcoidosis. The majority of COPD and asthma studies that measured CC16 in serum have reported lower levels compared to the control groups [62,63,64,65,66,67]. In contrast, the serum levels of CC16 in pulmonary fibrosis, sarcoidosis, and ARDS were higher in most patients [67,68,69,70,71,72]. CC16 levels in serum were also higher in patients with respiratory infections, such as respiratory syncytial virus [73]. In general, it appears that most patients with pulmonary diseases that predominantly affect the bronchial tubes (where the Clara cells are mainly located) tend to have low serum concentrations of CC16 as compared to healthy controls. Specifically, this can be seen in COPD and asthma studies. In contrast, patients with conditions characterized by inflammatory damage involving the alveolar-capillary barrier were found to have high serum levels of CC16, as shown in pulmonary fibrosis and sarcoidosis.

A recent study showed that patients infected with COVID-19 have diffuse alveolar damage [74]. Moreover, CC16 mRNA levels appeared to be dramatically reduced in the lungs of COVID-19 patients, and the circulating CC16 levels in serum were significantly decreased in COVID-19 patients compared to the healthy controls [75]. It was reported that Clara cells are involved in the repair of damaged epithelium [76]. Clara cells involved in wound repair become activated after alveolar injury. In injury remodeling, previous studies have found that Clara cells can migrate and restore injured alveoli in the lung [77,78,79]. Severe damage of lung epithelium may lead to a lower level of CC16 in circulation. Yin and colleagues observed that the number of CC16-positive Clara cells was significantly lower in the COVID-19 patient than in healthy controls [75]. Thus, there is a high possibility that SARS-CoV-2 causes severe damage to the Clara cells, which leads to reduced CC16 in COVID-19 patients. Therefore, CC16 might be a useful lung biomarker for respiratory complications of COVID-19 as well. However, more studies are needed to test the prognostic value of CC16 in COVID-19 patients. Currently, a pilot clinical trial is ongoing at the University of Arizona’s Asthma & Airways Disease Research Center, which aims to investigate the relationship between circulating CC16 and the clinical progression of COVID-19.

## 5. Discussion

Currently, multiple biomarkers in COVID-19 have been identified, such as leukocytes, C-reactive protein (CRP), procalcitonin (PCT), lactate dehydrogenase (LDH), ferritin, and cytokines (IL-2R, IL-6, IL-8, IL-10, and TNF-α) [80]. These biomarkers could potentially help clinicians diagnose and manage patients with COVID-19, and improve patients’ prognosis and outcomes. In our opinion, these biomarkers could be useful for assessing acute conditions in general, but not for the long-term impact of COVID-19 on the lung after hospital discharge. Compared to these biomarkers, circulating pnemoproteins in COVID-19 patients may reflect the lung condition, especially the injury of the lung epithelium. Thus, we reviewed the current knowledge of pneumoproteins and discussed their potential to serve as biomarkers for lung injury caused by COVID-19. The majority of these studies support the potential usefulness of pneumoproteins to assess COVID-19 patients’ prognosis. Still, additional studies are needed to confirm these observations and provide reliable and reproducible information for the benefit of patients.

One important limitation of this review is that the summarized studies are retrospective, with small sample sizes, and were conducted at one center. Large prospective cohort studies are needed to validate the current findings. Nevertheless, summarizing these small-scale studies could help clinicians and researchers conduct further research to address the unresolved matters.

## 6. Conclusions

In this review, retrospective and prospective studies focusing on the altered pneumoproteins levels in COVID-19 patients were summarized. Most studies included here aimed to evaluate KL-6 in response to COVID-19. Many ILDs have had KL-6 measured, and comparable findings between the studies have allowed for KL-6 to be a reliable biomarker for lung fibrosis. Two additional studies measured SP-D in coronavirus-infected individuals, and there is one ongoing clinical trial on CC16. To the best of our knowledge, no pneumoproteins other than these three have been reported so far. The current findings indicate KL-6, SP-D, and CC16 may reflect the severity of respiratory manifestations and could serve as potential biomarkers in COVID-19-related lung injury. Further investigations of these pneumoproteins in the circulation from COVID-19 patients are needed in the future.

## Figures and Tables

**Table 1:** The changes of KL-6 and SP-D levels in COVID-19 patients.

Reference	Protein	Country	No. of Patients	Conclusion	*p*-Value
[25]	KL-6	Italy	22 patients 22 healthy volunteers	KL-6 was higher in severe subjects than the non-severe subjects KL-6 was only elevated in the severe group admitted to the ICU and requiring mechanical ventilation	0.0118
				No significant difference in KL-6 level between non-severe COVID-19 cases and healthy controls	0.5277
[26]	KL-6	Belgium	83 COVID patients 70 healthy subjects 31 ILDs patients	Higher in COVID-19 cases than healthy subjects Lower in COVID-19 cases than ILD group	<0.001
				KL-6 was not associated with severe dyspnea	0.585
				KL-6 was not associated with ICU admission	0.434
				KL-6 did not show an impact on mortality	>0.05
				No correlation between high KL-6 levels and CRP	0.482
				High KL-6 was correlated with high LDH levels (*r* = 0.31)	0.004
				High-KL-6 levels were associated with higher values of platelet/lymphocyte ratio	0.04
[27]	KL-6	China	63 COVID patients 43 non-COVID patients	KL-6 was higher in COVID-19 than those in non-COVID-19 patients	<0.001
				KL-6 was higher in severe patients compared with mild patients	<0.05
				A significant correlation between KL-6 and pulmonary lesion area in severe cases (*r* = −0.14)	<0.05
				A significant correlation between the KL-6 and T lymphocyte (CD3+CD45+) in severe subjects (*r* = −0.24)	<0.05
				Ts (CD3+CD8+) and Th (CD3+CD4+) lymphocyte subsets were closely associated with KL-6 levels (*r* = −0.19 and −0.25)	<0.05
				IL-6 and IL-10 were significantly correlated with KL-6 levels, (*r* = 0.38 and 0.19)	<0.05
[28]	KL-6	Japan	21 severe COVID patients 33 non-severe patients	KL-6 was higher in the severe group than the non-severe group at admission and one week later	<0.001
[29]	KL-6	Japan, China; from multinational database	74 patients	KL-6 was associated with CT infiltrates	0.02
[30]	KL-6	Italy	26 patients	KL-6 levels were high at hospitalization and reduced after months of recovery	<0.05
				Increased in fibrotic than non-fibrotic group	0.0225
				In the fibrotic group, KL-6 reduced after 6 and 9 months of discharge	<0.05
[34]	KL-6	China	32 patients 7 healthy controls	KL-6 was higher compared to healthy controls	<0.05
[31]	KL-6	China	166 patients 59 healthy controls	KL-6 was higher compared to healthy controls	<0.001
				No significant correlation between KL-6 and pulmonary lesion area at the first week	>0.05
				A significant correlation between KL-6 and pulmonary lesion areas two weeks later	<0.001
				KL-6 gradually reduced after reaching a peak level in a month in some patients	<0.05
				A significant positive correlation between the serum KL-6 and CD4+CXCR5+ T cells (*r* = 0.535) and CD4+/CD8+ ratio (*r* = 0.511), and CD3+CD4+ T cells (*r* = 0.510) among severe patients	<0.05
				A significant negative correlation between the serum KL-6 and Tregs (*r* = −0.516), CD3+CD8+ T cells (*r* = −0.475), and CD8+CD161+ T cells (*r* = −0.425) among severe patients	<0.006
				KL-6 was correlated to coagulation indexes; d-dimer (*r* = 0.692) and fibrin degradation product FDP (*r* = 0.641) among severe patients	0.001
[35]	KL-6	Italy	54 patients	KL-6 higher in severe cases than in non-severe subjects	<0.0001
[36]	KL-6	Italy	34 patients	KL-6 higher in non-survivors compared to survivors	<0.001
				KL-6 above 1000 U/mL was independently associated with mortality compared to the P/F ratio and IL-6	<0.05
				KL-6 negatively correlated with the P/F ratio (*r* = 0.113)	<0.05
[37]	KL-6	Italy	41 patients 30 healthy controls	Serum concentrations of KL-6 were correlated with CRP (*r* = 0.51)	0.04
				Serum concentrations of KL-6 were correlated with IL-6 (*r* = 0.43)	0.04
				Peripheral levels of platelets showed an indirect correlation with KL-6 (*r* = −0.56)	0.04
[33]	KL-6	China	113 patients 36 suspected cases 65 healthy subjects	A significant positive correlation between the serum KL-6 and CRP levels in severe COVID-19 patients (*r* = 0.3803)	<0.001
				A significant negative correlation between the serum KL-6 and lymphocytes counts in severe COVID-19 patients (*r* = 0.1753)	0.0099
[38]	SP-D	Turkey	88 patients 20 healthy controls	Higher SP-D levels than the control group at admission	0.001
				No significant difference in SP-D levels between patients and controls at day 5	>0.05
				Higher SP-D levels in patients who developed ARDS or MAS compared to those who did not at admission and five days later	<0.05
				Higher among non-survivors compared to survivors	0.03
				A negative correlation between SP-D and PaO_2_/FiO_2_ level (*r* = −0.364)	0.01
[39]	SP-D	China	16 SARS patients 19 CAP patients 16 healthy controls	Higher SP-D levels in SARS patients than control	0.026
				No significant difference in SP-D levels between SARS patients and CAP patients	0.360

**Table 2:** Serum KL-6 and SP-D Concentrations in COVID-19.

Reference	Protein	Concentration	*p*-Value
[25]	KL-6	Severe cases (*n* = 12): 1021 (473–1909) U/mL Non-severe cases (*n* = 10): 293 (197–362) U/mL	0.0118
		Severe cases (*n* = 12): 1021 (473–1909) U/mL Healthy controls (*n* = 22): 239 (132–371) U/mL	0 .012
		Healthy controls (*n* = 22): 239 (132–371) U/mL Non-severe cases (*n* = 10): 293 (197–362) U/mL	0.5277
[26]	KL-6	Healthy subjects (*n* = 70): 254 (191–308) U/mL COVID-19 (*n* = 83): 405 (277–592) U/mL	< 0.001
		Healthy subjects (*n* = 70): 254 (191–308) U/mL Patients with interstitial lung diseases (*n* = 31): 897 (550–1885) U/mL	< 0.001
		Interstitial lung diseases (*n* = 31): 897 (550–1885) U/mL COVID-19 (*n* = 83): 405 (277–592) U/mL	< 0.001
[27]	KL-6	Non-COVID-19 patients (*n* = 43): 173.9 ± 63.40 U/mL COVID-19 patients (*n* = 63): Mild (*n* = 30): 241.2 ± 207.90; Severe (*n* = 33): 676.6 ± 506.70 U/mL	<0.001
		All patients (*n* = 63): Mild COVID-19 patients: 241.2 ± 207.90 U/mL Severe COVID-19 patients: 676.6 ± 506.70 U/mL	<0.001
[28]	KL-6	At diagnosis: Non-severe group (*n* = 33): 223 (166–255) U/mL Severe group (*n* = 21): 338 (303–529) U/mL	<0.001
		One week after diagnosis (peak levels): Non-severe group (*n* = 33): 234 (194–282) U/mL Severe group (*n* = 21): 781 (429–1435) U/mL	<0.001
[29]	KL-6	Patients with CT infiltrates (*n* = 48): 337 ± 173 U/mL Patients without CT infiltrates (*n* = 26): 227 ± 71 U/mL	0.021
[30]	KL-6	All patients (*n* = 26): At admission: 760 (311–1218) U/mL After 6 months: 309 (210–408) U/mL	0.0208
		All patients (*n* = 26): At admission: 760 (311–1218) U/mL After 9 months: 324 (279–458) U/mL	0.0365
		At admission: Fibrotic patients (*n* = 14): 755 (370–1023) U/mL Non-fibrotic patients (*n* = 12): 305 (225–608) U/mL	0.0225
		After 6 months: Fibrotic patients (*n* = 14): 290 (197–521) U/mL Non-fibrotic patients (*n* = 12): 262 (167–382) U/mL	0.2236
		After 9 months: Fibrotic patients (*n* = 14): 318 (173–435) U/mL Non-fibrotic patients (*n* = 12): 320 (214–427) U/mL	0.2536
		Fibrotic patients at admission: 755 (370–1023) U/mL Fibrotic patients after 6 months: 290 (197–521) U/mL	0.0366
		Fibrotic patients at admission: 755 (370–1023) U/mL Fibrotic patients after 9 months: 318 (173–435) U/mL	0.0490
[31]	KL-6	Severe patients: (*n* = 17): 898 (567.7–1278.9) U/mL Mild patients: (*n* = 149): 452.1 (325.6–641.3) U/mL Healthy subjects: (*n* = 59): 180.9 U/mL	<0.001
[35]	KL-6	Severe patients: (*n* = 14): 1125 (495–2034) U/mL Non-severe patients: (*n* = 40): 316 (210–398) U/mL	<0.0001
[36]	KL-6	At the time of enrollment (*n* = 34): 411 (177–1192) U/mL 7 days after enrollment (*n* = 34): 570 (70–7580) U/mL 14 days after enrollment (*n* = 15): 296 (137–5548) U/mL	-
		Patients with favorable outcome: (*n* = 19): 260 (125–421) U/mL Patients with unfavorable outcome: (*n* = 15): 1188 (592–3608) U/mL	<0.001
[37]	KL-6	Mild to moderate group (*n* = 14): 320 (226.3–927.8) U/mL Severe group (*n* = 10): 903 (333.8–1956) U/mL	0.035
[33]	KL-6	Control subjects (*n* = 65): 240.5 (217.5–285.5) U/mL severe group(*n* = 36) 373.7 (269.9–428.1) U/mL	<0.001
[38]	SP-D	Non-survivors: 96.7 ± 37.2 Survivors: 56.9 ± 43.5 ng/ml	0.03
		At admission COVID-19 patients with MAS (*n* = 20): 80.9 ± 45.5 ng/mL COVID-19 patients without MAS (*n* = 68): 53.7 ± 42.2 ng/mL Control (*n* = 20): 21.1 ± 18.6 ng/ml	0.001
		At admission COVID-19 patients with ARDS (*n* = 35): 82.3 ± 45.4 ng/mL COVID-19 patients without ARDS (*n* = 53): 46.5 ± 39.2 ng/mL Control (*n* = 20): 21.1 ± 18.6 ng/ml	0.001
		On day 5 COVID-19 patients with MAS (*n* = 20): 50.4 ± 18.3 ng/mL COVID-19 patients without MAS (*n* = 68): 35.6 ± 8.4 ng/ml	0.001
		On day 5 COVID-19 patients with ARDS (*n* = 35): 46.4 ± 33.2 ng/mL COVID-19 patients without ARDS (*n* = 53): 22.4 ± 18.9 ng/ml	0.001
[39]	SP-D	SARS patients (*n* = 16): 453 (379–963) ng/mL Control (*n* = 16): 218 (160–362) ng/ml	0.026
		SARS patients (*n* = 16): 453 (379–963) ng/mL CAP patients (*n* = 19): 302 (94–459) ng/ml	0.360

**Table 3:** Receiver-operating characteristic (ROC) analysis of the KL-6 levels from previous studies.

Reference	Aim	AUC%	Sensitivity %	Specificity %	Cut-Off Value U/mL	*p* Value
[25]	To evaluate disease severity	82.4 (95% CI: 62–100)	83	89	406.5	0.0129
[28]	To evaluate disease severity	At diagnosis: 84	76.2	86.2	303	<0.05
		One week after diagnosis 95	85.7	96.6	371	<0.05
[31]	To evaluate disease severity	79.3 (95% CI: 71.8–86.8)	75.3	73.3	642.3	<0.001
[29]	To determine asymptomatic patients with CT infiltrates	75 (58–91)	73	67	216	<0.05
[30]	To identify patients with fibrotic interstitial lung abnormalities	85 (95% CI: 64–100)	75	80	455	0.0404
[36]	To predict the critical outcome	84.9 (95% CI: 70.2–99.6)	-	-	1000	<0.01
[33]	To evaluate the severity of lung injury	82.66	80	68.13	278.3	<0.001

## Data Availability

No further or supplementary data is stated in the study.

## References

[1] Lu H., Stratton C.W., Tang Y.W. (2020). Outbreak of pneumonia of unknown etiology in Wuhan, China: The mystery and the miracle. J. Med. Virol..

[2] Cucinotta D., Vanelli M. (2020). WHO Declares COVID-19 a Pandemic. Acta Biomed..

[3] Fehr A.R., Perlman S. (2015). Coronaviruses: An overview of their replication and pathogenesis. Methods Mol. Biol..

[4] Cleri D.J., Ricketti A.J., Vernaleo J.R. (2010). Severe acute respiratory syndrome (SARS). Infect. Dis. Clin. N. Am..

[5] Bleibtreu A., Bertine M., Bertin C., Houhou-Fidouh N., Visseaux B. (2020). Focus on Middle East respiratory syndrome coronavirus (MERS-CoV). Med. Mal. Infect..

[6] Zhao S., Zhuang Z., Ran J., Lin J., Yang G., Yang L., He D. (2020). The association between domestic train transportation and novel coronavirus (2019-nCoV) outbreak in China from 2019 to 2020: A data-driven correlational report. Travel. Med. Infect. Dis..

[7] Chan J.F., Yuan S., Kok K.H., To K.K., Chu H., Yang J., Xing F., Liu J., Yip C.C., Poon R.W. (2020). A familial cluster of pneumonia associated with the 2019 novel coronavirus indicating person-to-person transmission: A study of a family cluster. Lancet.

[8] Huang C., Wang Y., Li X., Ren L., Zhao J., Hu Y., Zhang L., Fan G., Xu J., Gu X. (2020). Clinical features of patients infected with 2019 novel coronavirus in Wuhan, China. Lancet.

[9] Whittle J.S., Pavlov I., Sacchetti A.D., Atwood C., Rosenberg M.S. (2020). Respiratory support for adult patients with COVID-19. J. Am. Coll. Emerg. Physicians Open.

[10] Rodriguez-Morales A.J., Cardona-Ospina J.A., Gutierrez-Ocampo E., Villamizar-Pena R., Holguin-Rivera Y., Escalera-Antezana J.P., Alvarado-Arnez L.E., Bonilla-Aldana D.K., Franco-Paredes C., Henao-Martinez A.F. (2020). Clinical, laboratory and imaging features of COVID-19: A systematic review and meta-analysis. Travel. Med. Infect. Dis..

[11] Cao Y., Liu X., Xiong L., Cai K. (2020). Imaging and clinical features of patients with 2019 novel coronavirus SARS-CoV-2: A systematic review and meta-analysis. J. Med. Virol..

[12] Fraser E. (2020). Long term respiratory complications of COVID-19. BMJ.

[13] Ngai J.C., Ko F.W., Ng S.S., To K.W., Tong M., Hui D.S. (2010). The long-term impact of severe acute respiratory syndrome on pulmonary function, exercise capacity and health status. Respirology.

[14] Zhang P., Li J., Liu H., Han N., Ju J., Kou Y., Chen L., Jiang M., Pan F., Zheng Y. (2020). Long-term bone and lung consequences associated with hospital-acquired severe acute respiratory syndrome: A 15-year follow-up from a prospective cohort study. Bone Res..

[15] Hu Y., Wang L.S., Jin Y.P., Du S.S., Du Y.K., He X., Weng D., Zhou Y., Li Q.H., Shen L. (2017). Serum Krebs von den Lungen-6 level as a diagnostic biomarker for interstitial lung disease in Chinese patients. Clin. Respir. J..

[16] Ohshimo S., Yokoyama A., Hattori N., Ishikawa N., Hirasawa Y., Kohno N. (2005). KL-6, a human MUC1 mucin, promotes proliferation and survival of lung fibroblasts. Biochem. Biophys. Res. Commun..

[17] Hanson K.M., Hernady E.B., Reed C.K., Johnston C.J., Groves A.M., Finkelstein J.N. (2019). Apoptosis Resistance in Fibroblasts Precedes Progressive Scarring in Pulmonary Fibrosis and Is Partially Mediated by Toll-Like Receptor 4 Activation. Toxicol. Sci..

[18] Kuwana M., Shirai Y., Takeuchi T. (2016). Elevated Serum Krebs von den Lungen-6 in Early Disease Predicts Subsequent Deterioration of Pulmonary Function in Patients with Systemic Sclerosis and Interstitial Lung Disease. J. Rheumatol..

[19] Jiang Y., Luo Q., Han Q., Huang J., Ou Y., Chen M., Wen Y., Mosha S.S., Deng K., Chen R. (2018). Sequential changes of serum KL-6 predict the progression of interstitial lung disease. J. Thorac. Dis..

[20] Yokoyama A., Kondo K., Nakajima M., Matsushima T., Takahashi T., Nishimura M., Bando M., Sugiyama Y., Totani Y., Ishizaki T. (2006). Prognostic value of circulating KL-6 in idiopathic pulmonary fibrosis. Respirology.

[21] Sato H., Callister M.E., Mumby S., Quinlan G.J., Welsh K.I., duBois R.M., Evans T.W. (2004). KL-6 levels are elevated in plasma from patients with acute respiratory distress syndrome. Eur. Respir. J..

[22] Inoue Y., Nishimura K., Shiode M., Akutsu H., Hamada H., Fujioka S., Fujino S., Yokoyama A., Kohno N., Hiwada K. (1995). Evaluation of serum KL-6 levels in patients with pulmonary tuberculosis. Tuber Lung Dis..

[23] Dilli D., Ozyazici A., Dursun A., Beken S. (2017). Predictive values of plasma KL-6 in bronchopulmonary dysplasia in preterm infants. Turk. J. Med. Sci..

[24] Song P., Li W., Xie J., Hou Y., You C. (2020). Cytokine storm induced by SARS-CoV-2. Clin. Chim. Acta.

[25] d’Alessandro M., Cameli P., Refini R.M., Bergantini L., Alonzi V., Lanzarone N., Bennett D., Rana G.D., Montagnani F., Scolletta S. (2020). Serum KL-6 concentrations as a novel biomarker of severe COVID-19. J. Med. Virol..

[26] Frix A.N., Schoneveld L., Ladang A., Henket M., Duysinx B., Vaillant F., Misset B., Moutschen M., Louis R., Cavalier E. (2020). Could KL-6 levels in COVID-19 help to predict lung disease?. Respir. Res..

[27] Xue M., Zheng P., Bian X., Huang Z., Huang H., Zeng Y., Hu H., Liu X., Zhou L., Sun B. (2020). Exploration and correlation analysis of changes in Krebs von den Lungen-6 levels in COVID-19 patients with different types in China. Biosci. Trends.

[28] Awano N., Inomata M., Kuse N., Tone M., Takada K., Muto Y., Fujimoto K., Akagi Y., Mawatari M., Ueda A. (2020). Serum KL-6 level is a useful biomarker for evaluating the severity of coronavirus disease 2019. Respir. Investig..

[29] Varble N., Blain M., Kassin M., Xu S., Turkbey E.B., Amalou A., Long D., Harmon S., Sanford T., Yang D. (2021). CT and clinical assessment in asymptomatic and pre-symptomatic patients with early SARS-CoV-2 in outbreak settings. Eur. Radiol..

[30] d’Alessandro M., Bergantini L., Cameli P., Curatola G., Remediani L., Bennett D., Bianchi F., Perillo F., Volterrani L., Mazzei M.A. (2021). Serial KL-6 measurements in COVID-19 patients. Intern. Emerg. Med..

[31] Deng K., Fan Q., Yang Y., Deng X., He R., Tan Y., Lan Y., Deng X., Pan Y., Wang Y. (2021). Prognostic roles of KL-6 in disease severity and lung injury in COVID-19 patients: A longitudinal retrospective analysis. J. Med. Virol..

[32] Ding M., Zhang Q., Li Q., Wu T., Huang Y.Z. (2020). Correlation analysis of the severity and clinical prognosis of 32 cases of patients with COVID-19. Respir. Med..

[33] Peng D.H., Luo Y., Huang L.J., Liao F.L., Liu Y.Y., Tang P., Hu H.N., Chen W. (2021). Correlation of Krebs von den Lungen-6 and fibronectin with pulmonary fibrosis in coronavirus disease 2019. Clin. Chim Acta.

[34] Zeng H.L., Chen D., Yan J., Yang Q., Han Q.Q., Li S.S., Cheng L. (2021). Proteomic characteristics of bronchoalveolar lavage fluid in critical COVID-19 patients. FEBS J..

[35] d’Alessandro M., Bergantini L., Cameli P., Curatola G., Remediani L., Sestini P., Bargagli E., Siena C.U. (2021). Peripheral biomarkers’ panel for severe COVID-19 patients. J. Med. Virol..

[36] Scotto R., Pinchera B., Perna F., Atripaldi L., Giaccone A., Sequino D., Zappulo E., Sardanelli A., Schiano Moriello N., Stanziola A. (2021). Serum KL-6 Could Represent a Reliable Indicator of Unfavourable Outcome in Patients with COVID-19 Pneumonia. Int. J. Environ. Res. Public Health.

[37] Bergantini L., Bargagli E., d’Alessandro M., Refini R.M., Cameli P., Galasso L., Scapellato C., Montagnani F., Scolletta S., Franchi F. (2021). Prognostic bioindicators in severe COVID-19 patients. Cytokine.

[38] Kerget B., Kerget F., Kocak A.O., Kiziltunc A., Araz O., Ucar E.Y., Akgun M. (2020). Are Serum Interleukin 6 and Surfactant Protein D Levels Associated with the Clinical Course of COVID-19?. Lung.

[39] Wu Y.P., Liu Z.H., Wei R., Pan S.D., Mao N.Y., Chen B., Han J.J., Zhang F.S., Holmskov U., Xia Z.L. (2009). Elevated plasma surfactant protein D (SP-D) levels and a direct correlation with anti-severe acute respiratory syndrome coronavirus-specific IgG antibody in SARS patients. Scand. J. Immunol..

[40] Lopez-Rodriguez E., Gay-Jordi G., Mucci A., Lachmann N., Serrano-Mollar A. (2017). Lung surfactant metabolism: Early in life, early in disease and target in cell therapy. Cell Tissue Res..

[41] Han S., Mallampalli R.K. (2015). The Role of Surfactant in Lung Disease and Host Defense against Pulmonary Infections. Ann. Am. Thorac. Soc..

[42] Vieira F., Kung J.W., Bhatti F. (2017). Structure, genetics and function of the pulmonary associated surfactant proteins A and D: The extra-pulmonary role of these C type lectins. Ann. Anat..

[43] Singh G., Katyal S.L. (1997). Clara cells and Clara cell 10 kD protein (CC10). Am. J. Respir. Cell Mol. Biol..

[44] Fehrenbach H. (2001). Alveolar epithelial type II cell: Defender of the alveolus revisited. Respir Res..

[45] Walther F.J., Waring A.J., Sherman M.A., Zasadzinski J.A., Gordon L.M. (2007). Hydrophobic surfactant proteins and their analogues. Neonatology.

[46] Sorensen G.L., Husby S., Holmskov U. (2007). Surfactant protein A and surfactant protein D variation in pulmonary disease. Immunobiology.

[47] Kishore U., Greenhough T.J., Waters P., Shrive A.K., Ghai R., Kamran M.F., Bernal A.L., Reid K.B., Madan T., Chakraborty T. (2006). Surfactant proteins SP-A and SP-D: Structure, function and receptors. Mol. Immunol..

[48] Parra E., Alcaraz A., Cruz A., Aguilella V.M., Perez-Gil J. (2013). Hydrophobic pulmonary surfactant proteins SP-B and SP-C induce pore formation in planar lipid membranes: Evidence for proteolipid pores. Biophys. J..

[49] Griese M., Lorenz E., Hengst M., Schams A., Wesselak T., Rauch D., Wittmann T., Kirchberger V., Escribano A., Schaible T. (2016). Surfactant proteins in pediatric interstitial lung disease. Pediatr. Res..

[50] Mulugeta S., Beers M.F. (2006). Surfactant protein C: Its unique properties and emerging immunomodulatory role in the lung. Microbes Infect..

[51] Khoor A., Stahlman M.T., Gray M.E., Whitsett J.A. (1994). Temporal-spatial distribution of SP-B and SP-C proteins and mRNAs in developing respiratory epithelium of human lung. J. Histochem. Cytochem..

[52] Rao M.L., Rao G.S., Eckel J., Breuer H. (1977). Factors involved in the uptake of corticosterone by rat liver cells. Biochim. Biophys. Acta.

[53] Doyle I.R., Bersten A.D., Nicholas T.E. (1997). Surfactant proteins-A and -B are elevated in plasma of patients with acute respiratory failure. Am. J. Respir. Crit. Care Med..

[54] Ohlmeier S., Vuolanto M., Toljamo T., Vuopala K., Salmenkivi K., Myllarniemi M., Kinnula V.L. (2008). Proteomics of human lung tissue identifies surfactant protein A as a marker of chronic obstructive pulmonary disease. J. Proteome Res..

[55] Greene K.E., King T.E., Kuroki Y., Bucher-Bartelson B., Hunninghake G.W., Newman L.S., Nagae H., Mason R.J. (2002). Serum surfactant proteins-A and -D as biomarkers in idiopathic pulmonary fibrosis. Eur. Respir. J..

[56] Yi Y., Lagniton P.N.P., Ye S., Li E., Xu R.H. (2020). COVID-19: What has been learned and to be learned about the novel coronavirus disease. Int. J. Biol. Sci..

[57] Fujita M., Shannon J.M., Ouchi H., Voelker D.R., Nakanishi Y., Mason R.J. (2005). Serum surfactant protein D is increased in acute and chronic inflammation in mice. Cytokine.

[58] Eisner M.D., Parsons P., Matthay M.A., Ware L., Greene K., Acute Respiratory Distress Syndrome N. (2003). Plasma surfactant protein levels and clinical outcomes in patients with acute lung injury. Thorax.

[59] Determann R.M., Royakkers A.A., Haitsma J.J., Zhang H., Slutsky A.S., Ranieri V.M., Schultz M.J. (2010). Plasma levels of surfactant protein D and KL-6 for evaluation of lung injury in critically ill mechanically ventilated patients. BMC Pulm. Med..

[60] Klug J., Beier H.M., Bernard A., Chilton B.S., Fleming T.P., Lehrer R.I., Miele L., Pattabiraman N., Singh G. (2000). Uteroglobin/Clara cell 10-kDa family of proteins: Nomenclature committee report. Ann. N. Y. Acad. Sci..

[61] Broeckaert F., Bernard A. (2000). Clara cell secretory protein (CC16): Characteristics and perspectives as lung peripheral biomarker. Clin. Exp. Allergy.

[62] Park H.Y., Churg A., Wright J.L., Li Y., Tam S., Man S.F., Tashkin D., Wise R.A., Connett J.E., Sin D.D. (2013). Club cell protein 16 and disease progression in chronic obstructive pulmonary disease. Am. J. Respir. Crit. Care Med..

[63] Guerra S., Halonen M., Vasquez M.M., Spangenberg A., Stern D.A., Morgan W.J., Wright A.L., Lavi I., Tares L., Carsin A.E. (2015). Relation between circulating CC16 concentrations, lung function, and development of chronic obstructive pulmonary disease across the lifespan: A prospective study. Lancet Respir. Med..

[64] Lomas D.A., Silverman E.K., Edwards L.D., Miller B.E., Coxson H.O., Tal-Singer R. (2008). Evaluation of serum CC-16 as a biomarker for COPD in the ECLIPSE cohort. Thorax.

[65] Laing I.A., Hermans C., Bernard A., Burton P.R., Goldblatt J., Le Souef P.N. (2000). Association between plasma CC16 levels, the A38G polymorphism, and asthma. Am. J. Respir. Crit. Care Med..

[66] Oh J.Y., Lee Y.S., Min K.H., Hur G.Y., Lee S.Y., Kang K.H., Rhee C.K., Park S.J., Shim J.J. (2018). Decreased serum club cell secretory protein in asthma and chronic obstructive pulmonary disease overlap: A pilot study. Int. J. Chron. Obs. Pulmon. Dis..

[67] Almuntashiri S., Zhu Y., Han Y., Wang X., Somanath P.R., Zhang D. (2020). Club Cell Secreted Protein CC16: Potential Applications in Prognosis and Therapy for Pulmonary Diseases. J. Clin. Med..

[68] Lesur O., Langevin S., Berthiaume Y., Legare M., Skrobik Y., Bellemare J.F., Levy B., Fortier Y., Lauzier F., Bravo G. (2006). Outcome value of Clara cell protein in serum of patients with acute respiratory distress syndrome. Intensive Care Med..

[69] Lin J., Zhang W., Wang L., Tian F. (2018). Diagnostic and prognostic values of Club cell protein 16 (CC16) in critical care patients with acute respiratory distress syndrome. J. Clin. Lab. Anal..

[70] Buendia-Roldan I., Ruiz V., Sierra P., Montes E., Ramirez R., Vega A., Salgado A., Vargas M.H., Mejia M., Pardo A. (2016). Increased Expression of CC16 in Patients with Idiopathic Pulmonary Fibrosis. PLoS ONE.

[71] Kokuho N., Ishii T., Kamio K., Hayashi H., Kurahara M., Hattori K., Motegi T., Azuma A., Gemma A., Kida K. (2015). Diagnostic Values For Club Cell Secretory Protein (CC16) in Serum of Patients of Combined Pulmonary Fibrosis and Emphysema. COPD.

[72] Hermans C., Petrek M., Kolek V., Weynand B., Pieters T., Lambert M., Bernard A. (2001). Serum Clara cell protein (CC16), a marker of the integrity of the air-blood barrier in sarcoidosis. Eur. Respir. J..

[73] Johansson S., Kristjansson S., Bjarnarson S.P., Wennergren G., Rudin A. (2009). Clara cell protein 16 (CC16) serum levels in infants during respiratory syncytial virus infection. Acta Paediatr..

[74] Roden A.C., Bois M.C., Johnson T.F., Aubry M.C., Alexander M.P., Hagen C.E., Lin P.T., Quinton R.A., Maleszewski J.J., Boland J.M. (2021). The Spectrum of Histopathologic Findings in Lungs of Patients With Fatal Coronavirus Disease 2019 (COVID-19) Infection. Arch. Pathol. Lab. Med..

[75] Yin W., Cao W., Zhou G., Wang L., Sun J., Zhu A., Wang Z., Zhou Y., Liu X., Li Y. (2021). Analysis of pathological changes in the epithelium in COVID-19 patient airways. ERJ Open Res..

[76] Crosby L.M., Waters C.M. (2010). Epithelial repair mechanisms in the lung. Am. J. Physiol. Lung Cell Mol. Physiol..

[77] Fukuda Y., Takemura T., Ferrans V.J. (1989). Evolution of metaplastic squamous cells of alveolar walls in pulmonary fibrosis produced by paraquat. An ultrastructural and immunohistochemical study. Virchows Arch. B Cell Pathol. Incl. Mol. Pathol..

[78] Rock J.R., Barkauskas C.E., Cronce M.J., Xue Y., Harris J.R., Liang J., Noble P.W., Hogan B.L. (2011). Multiple stromal populations contribute to pulmonary fibrosis without evidence for epithelial to mesenchymal transition. Proc. Natl. Acad. Sci. USA.

[79] Fukumoto J., Soundararajan R., Leung J., Cox R., Mahendrasah S., Muthavarapu N., Herrin T., Czachor A., Tan L.C., Hosseinian N. (2016). The role of club cell phenoconversion and migration in idiopathic pulmonary fibrosis. Aging.

[80] Samprathi M., Jayashree M. (2020). Biomarkers in COVID-19: An Up-To-Date Review. Front. Pediatr..

